# Variation of Nonlinear Refraction and Three-Photon Absorption of Indium–Tin Oxide Quantum Dot Thin Films and Solutions in Near Infrared Range

**DOI:** 10.3390/nano13162320

**Published:** 2023-08-12

**Authors:** Arturs Bundulis, Anete Berzina, Vyacheslav V. Kim, Boris Polyakov, Aleksandrs Novikovs, Rashid A. Ganeev

**Affiliations:** 1 Institute of Solid State Physics, Kengaraga 8, LV-1063 Riga, Latvia; arturs.bundulis@cfi.lu.lv (A.B.); anete.berzina@cfi.lu.lv (A.B.); boris.polyakov@cfi.lu.lv (B.P.); aleksandrs.novikovs@cfi.lu.lv (A.N.); 2 Laboratory of Nonlinear Optics, University of Latvia, Jelgavas 3, LV-1586 Riga, Latvia; vyacheslav.kim@lu.lv; 3 Institute of Fundamental and Applied Research, TIIAME National Research University, Kori Niyoziy 39, Tashkent 100000, Uzbekistan; 4 Department of Physics and Chemistry, Chirchik State Pedagogical University, 104 Amir Temur, Chirchik 111700, Uzbekistan; 5 Department of Physics, Voronezh State University, Voronezh 394006, Russia

**Keywords:** indium–tin oxide, quantum dots, Kerr effect, three-photon absorption, third harmonic generation

## Abstract

We characterize the nonlinear optical properties of indium–tin oxide (ITO) quantum dots (QDs) in the IR range using the Z-scan method. We present results of three-photon absorption (3PA), third harmonic generation (3HG), and Kerr-effect-induced nonlinear refraction in ITO QDs. Z-scan measurements were carried out for the QDs solution, while 3HG was demonstrated using QD thin films. The Kerr-induced nonlinear refractive index was analyzed along the 800–950 nm range showing an increase in this parameter from −6.7 × 10^−18^ to −1.5 × 10^−17^ m^2^ W^−1^. At longer wavelengths (1000–1100 nm), the higher-order effects started to contribute to a nonlinear refractive index. The 3PA coefficient at 950 nm was measured to be 1.42 × 10^−25^ m^3^/W^2^. We discuss the peculiarities in the wavelength-dependent variation of the coefficient of nonlinear absorption responsible for 3PA in the range of 800–1150 nm. Third harmonic generation was analyzed in the 1200–1550 nm spectral range. The absolute value of 3HG conversion efficiency in the 150 nm thick film at the wavelength of laser radiation (1350 nm) was estimated to be ~10^–5^.

## 1. Introduction

The interest in small-sized structures is due to their potential application in various fields of science such as, in particular, nonlinear optics [1]. One such application can be the creation of media based on nanostructures for converting laser radiation into the extreme ultraviolet region of the spectrum. Other applications of nanoparticles (NPs) and quantum dots (QDs) may include optical limiters, as well as passive modulators and Q-switchers in laser oscillators based on the studies of the nonlinear optical properties of these structures [2,3,4,5,6].

Attention to the QDs based on various materials is also due to their practical applications in the processes of photosynthesis, as well as their sensors and biomarkers. An important aspect of the study, in this case, is the analysis of their nonlinear optical properties, in particular, two-photon absorption (2PA). Meanwhile, for the case of longer wavelengths of laser radiation (*λ* > 860 nm), one can expect a greater influence of three-photon absorption (3PA) in materials such as ZnS quantum dots, while at *λ* < 860 nm, a reverse saturable absorption (RSA) could play an important role. Another effect—third harmonic generation (3HG)—is also of interest in the case of such small-sized structures. The analysis of these processes allows us to determine the characteristics of monoatomic structures and NPs of various materials. One of these materials is indium–tin oxide.

Indium–tin oxide (ITO) is a widely studied material for various optoelectronic applications. It is a ternary composition of indium, tin, and oxygen in varying proportions. Depending on the oxygen content, it can be described as either a ceramic or an alloy. Indium–tin oxide is typically encountered as an oxygen-saturated composition with a formulation of 74% In, 18% Sn, and 8% O by weight. It is transparent and colorless in thin layers, while in bulk form it is yellowish to gray. In the infrared region of the spectrum, it acts as a metal-like mirror. Indium–tin oxide is one of the most widely used transparent conducting oxides. As with all transparent conducting films, a compromise must be made between conductivity and transparency, since increasing the thickness and increasing the concentration of charge carriers increases the film’s conductivity but decreases its transparency. 

ITO can be used in nanotechnology to provide a path to a new generation of solar cells. Solar cells made with these devices have the potential to provide low-cost, ultra-lightweight, and flexible cells with a wide range of applications. Because of the nanoscale dimensions of the nanorods, quantum-sized effects influence their optical properties. By tailoring the size of the rods, they can be made to absorb light within a specific narrow band of colors. By stacking several cells with different-sized rods, a broad range of wavelengths across the solar spectrum can be collected and converted to energy. Moreover, the nanoscale volume of the rods leads to a significant reduction in the amount of semiconductor material needed compared to a conventional cell. 

While there are numerous studies about ITO applications in optoelectronics (for example [7,8,9,10]), there is a limited amount of research regarding its nonlinear optical (NLO) properties and applications. In [11], the nonlinear optical properties of ITO thin film coated on soda–lime glass substrate were studied using the Z-scan technique. Nonlinear optical absorption showed a competition between saturable absorption (SA) and RSA at different excitation wavelengths and incident intensities. A transition from SA to RSA was observed when increasing the excitation wavelength. The measured nonlinear refractive index was found to be dependent on a wavelength with a maximum value of 5 × 10^−12^ cm^2^ W^−1^ at the wavelength of 900 nm. Recently, the influence of ITO thin-film thickness on the optical Kerr nonlinearity using ultrashort laser pulses was reported [12]. The effect of crystallinity on the nonlinear optical properties of ITO thin films was analyzed in [13]. 

The propagation of light in the epsilon-near-zero (ENZ) region of materials exhibits intriguing linear and nonlinear optical phenomena that have been extensively exploited. It was shown in [14] that the optical properties as well as the ENZ wavelength of magnetron-sputtered ITO thin films could be judiciously engineered. The measurement of nonlinear optical properties reveals that the control of deposition conditions allows for the tuning of absorptive optical nonlinearity between SA and RSA. The ENZ wavelength for the ITO film is deduced as around 1553 nm. The highest third-order nonlinear absorption coefficient and imaginary part of third-order nonlinear susceptibility for the ITO thin film through the Z-scan method were reported to be 5 × 10^−10^ m W^−1^ and 4 × 10^−14^ e.s.u. at 1050 nm and −6 × 10^−10^ m W^−1^ and 5 × 10^−14^ e.s.u. at 1550 nm, respectively [14]. These properties allow for the application of the ITO thin film for the Q-switched pulse laser generation in the ∼1050 and ∼1550 nm regions. Thus, due to the low refractive index of ITO thin films (|*εR*| < 1 in the range of 850–2060 nm), it is a promising material for the enhancement of NLO effects. 

While there are examples of research papers that have characterized third-order NLO properties of ITO structures at specific wavelengths in infrared and visible ranges, the studies regarding ITO QDs are even fewer with examples of Z-scan measurements at He-Ne laser wavelength [15]. Overall, there is a lack of studies on the NLO properties of ITO QDs in a broader spectral range. The significance and timeliness of these studies are related to the applications in the formation of efficient sources of coherent ultraviolet and extreme ultraviolet radiation, as well as the potential use of these nanostructured species in optics and optoelectronics. 

In this paper, we characterize the NLO properties of ITO QDs using the Z-scan method. We present the studies of the 3PA, 3HG, and Kerr-effect-induced response of ITO QDs. Z-scan measurements were carried out for the QDs solution with QDs dissolved in hexane. Thin ITO QD films were used to demonstrate 3HG. The Kerr-induced nonlinear refractive index was analyzed along the 800–950 nm range showing a slow increase in this parameter. We discuss the peculiarities in the wavelength-dependent variation of the coefficient of nonlinear absorption responsible for the 3PA in the range of 800–1150 nm. Third harmonic generation was analyzed in the 1200–1550 nm spectral range of probe pulses.

## 2. Description of Samples and Method of Measurements

ITO QDs were synthesized as follows: 20 mL of octadecene (ODE), 1 mL of oleic acid, 0.26 g of indium acetate, and 0.023 g of tin (II) chloride dihydrate were added to a 50 mL three-necked flask equipped with a water-cooled vertical quencher. The solution was degassed at 110 °C for 2 h under nitrogen flow and then continuously purged with nitrogen during the whole synthesis procedure. After that, the solution was heated to 295 °C until the color became yellow-orange. Then, an amine solution composed of oleylamine OLAM (2 mM) in 2mL of ODE was rapidly injected by a syringe into the flask to initiate particle nucleation. The temperature was decreased to 280 °C and kept constant for 2 h and then cooled down to room temperature. The color of the final solution was light green. 

**Figure 1:**
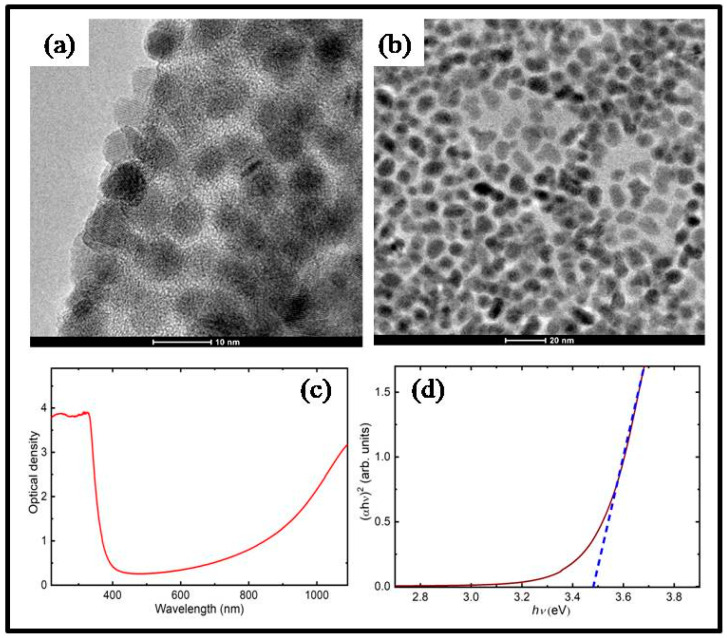
Characterization of ITO QDs. (**a**,**b**) TEM images of ITO QDs at different resolutions (scale bars 10 and 20 nm respectively). The mean size of particles was found to be 4 nm. (**c**) The absorption spectrum of ITO QD solution. (**d**) The Tauc plot demonstrates the band gap calculation of ITO QDs.

Product washing was carried out by standard polar/nonpolar solvent precipitation techniques utilizing a high-speed centrifuge. An equal amount of toluene and three volumes of acetone were added to three-necked flasks, poured into 50 mL centrifuge tubes, and centrifuged for 10 min at a speed of 6000 rpm. The washing cycles were repeated twice. Finally, ITO QDs were redispersed in toluene. TEM images of QDs are shown in Figure 1a,b. It can be seen that the mean size of the QDs is around 4 nm.

The absorption spectrum of the ITO QD solution is shown in Figure 1c. The synthesized QDs displayed strong absorption in the UV region, which was similar to the absorption spectrum of ITO nanocolloids of size 20–25 nm with a band gap energy of 3.73 eV [15]. Compared to other studies, these QDs showed larger absorption in the near-infrared region (i.e., at the wavelengths above 800 nm). Previous studies have shown that by decreasing the size of ITO QDs, the absorption band shifts closer to the visible range [16]. For example, the 9 nm ITO NPs showed an absorption maximum of around 1600 nm compared to the 30 nm NPs that had an absorption maximum at 2000 nm. The band gap energy for QDs was calculated using the Tauc relation [17], found to be 3.47 eV (Figure 1d).

The characterization of NLO properties was carried out using the standard Z-scan technique [18,19]. A tunable femtosecond laser source (ORPHEUS-HP + PHAROS PH2, Light Conversion) was used in a setup with 150 fs pulse duration and 500 kHz repetition rate with a tunable spectral range of 300–1700 nm. The laser beam was focused using the 110 mm focal length lens. The details of the used Z-scan configuration are presented elsewhere [20,21]. The 3HG was measured in the case of the femtosecond pulses tuned along the spectral range between 1100 nm and 1500 nm with a 50 nm step. The experimental setup was similar to the Z-scan, while the generated third harmonic was detected using the fiber optics spectrometer (Ocean Optics HR4000). 

## 3. Nonlinear Optical Measurements and Discussion

Kerr and two-photon absorption effects can be described using the following equations:

(1) $$\left\{\begin{array}{c} n=n_{0}+\gamma \cdot I\\ \alpha =\alpha_{0}+\beta_{2PA}\cdot I \end{array}\right.\;.\;$$

Here, *I* is an intensity of the probe pulses, *n*_0_ and *α*_0_ are the linear refractive index and absorption, *γ* is a Kerr-induced nonlinear refractive index, and *β*_2*PA*_ is a 2PA coefficient. To simultaneously study 2PA and Kerr effects, the open-aperture and closed-aperture schemes of the Z-scan were used. A detector in the case of an open-aperture scheme measures the overall power variation of the transmitted beam to determine the 2PA effect. The closed-aperture measurement can detect both effects (nonlinear absorption and nonlinear refraction) simultaneously. To separate the Kerr effect contribution from the 2PA, the closed-aperture data were divided by the open-aperture data. The influence of the Kerr effect on the closed-aperture measurements can be expressed as:

(2) $$T_{CA}=1+\frac{4\cdot ∆\Phi \cdot \frac{z}{z_{R}}}{\left({\left(\frac{z}{z_{R}}\right)}^{2}+9\right)\left({\left(\frac{z}{z_{R}}\right)}^{2}+1\right)}\;,\;$$

where *T_CA_* is the normalized transmittance of the closed-aperture configuration, *z* is a sample position relative to the focal point, *z_R_* is a Rayleigh length, and ΔΦ is defined as:

(3) $$∆\Phi =k\cdot \gamma \cdot L_{eff}\cdot I\;.\;$$

Here, *k* is a wave number of the probe radiation, and *L_eff_* is an effective thickness of the studied sample defined as:

(4) $$L_{eff}=\frac{1-e^{-\alpha_{0}\cdot L}}{\alpha_{0}}\;,\;$$

where *L* is a sample thickness. 

The influence of 3PA on the open-aperture measurement can be described using the equation:

(5) $$T_{OA}=\frac{{sinh}^{-1}\left(\frac{\Psi }{{\left(\frac{z}{z_{R}}\right)}^{2}+1}\right)}{\frac{\Psi }{{\left(\frac{z}{z_{R}}\right)}^{2}+1}}\;,\;$$

where Ψ is defined as:

(6) $$\Psi =\sqrt{2\cdot \beta_{3PA}\cdot L_{eff}\cdot I^{2}}\;$$

Here, *β*_3*PA*_ is a 3PA coefficient.

Below, we present the results of the studies of 3PA, 3HG, and Kerr effects during propagation of the focused 150 fs pulses of different wavelengths through the films and solutions containing ITO QDs. Z-scan measurements were carried out for the QDs solution, with QDs dissolved in hexane. The solution was contained in a 1 mm thick quartz cell. Third harmonic generation measurements were carried out using thin films containing ITO QDs and deposited on the 1 mm thick fused silica substrate. The thin films of 150 nm thickness were produced using the blade-casting method. To account for substrate contribution, parallel to the thin-film measurements, a third harmonic from the pure substrate was also measured.

### 3.1. Three-Photon Absorption

**Figure 2:**
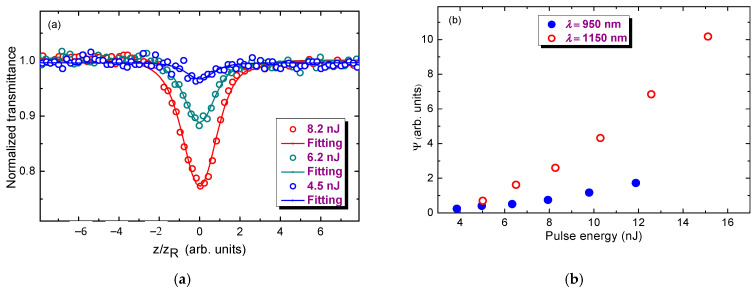
(**a**) Open-aperture measurements of the ITO QDs solution at *λ* = 950 nm at different pulse energies fitted with Equation (5). (**b**) Dependencies of the parameter Ψ determining *β*_3*PA*_ on the power of probe pulses at two wavelengths (950 and 1150 nm).

ITO QDs exhibited strong 3PA in the case of IR pulses. This process dominated in the case of the Z-scans in the spectral range of 800–1150 nm. The example of open-aperture Z-scan data using the 950 nm probe pulses (*E*_ph_ = 1.3 eV) is shown in Figure 2a for three average powers of the 500 kHz probe pulses. In all cases, the Rayleigh length was equal to 1.2 mm. The open-aperture Z-scans of the ITO QDs solution at different pulse energies (4.5, 6.2, and 8.3 nJ) were fitted using Equation (5), thus confirming that the observed nonlinear optical process is attributed to the simultaneous absorption of three probe photons rather than 2PA. Indeed, the probability of 2PA using 1.3 eV photons is rather insignificant in the QDs with a band gap of 3.47 eV. Correspondingly, 2PA can start to play an important role at larger energies of the probe pulses (*E*_ph_ > 1.7 eV, *λ* < 730 nm). 

ITO has a large absorption band around 3.47 eV (see Figure 1c), thus indicating that the measurements at 950 nm (*E*_ph_ ≈ 1.3 eV) should correspond to the 3PA process. The dependencies of the parameter Ψ determining the coefficient of three-photon absorption on the laser power are shown in Figure 2b for two wavelengths (950 and 1150 nm). The graph measured using 950 nm pulses demonstrates almost linear Ψ(*I*) dependence with a corresponding three-photon absorption coefficient of *β*_3*PA*;950_ = 1.42 ∙ 10^−25^ m^3^/W^2^. 

In the case of 1150 nm, one can assume stronger dependence of Ψ on the energy of probe pulses. Looking at Equation (6), one can assume that parameter Ψ linearly depends on the pulse intensity and correspondingly the energy of probe pulses, which corroborates the Ψ(*I*) curve observed in the case of 950 nm pulses (Figure 2b, blue filled circles). The application of longer wavelength pulses (*λ* = 1150 nm, *E*_ph_ = 1.08 eV) decreases the probability of the 3PA process in these QDs, thus assuming that another, higher-order nonlinear optical process like four-photon absorption starts playing the measurable role in this medium. Correspondingly, the dependence Ψ(*I*) becomes rather nonlinear once one assumes the replacement of *β*_3*PA*_ by the next order of nonlinearity (four-photon absorption, *β*_4*PA*_ × *I*). In that case, the expected dependence of Ψ(*I*) will be equal to Ψ ∝ *I*^3/2^ (see Equation (6)), which is close to the experimental plot shown in Figure 2b for the 1150 nm probe wavelength. 

**Figure 3:**
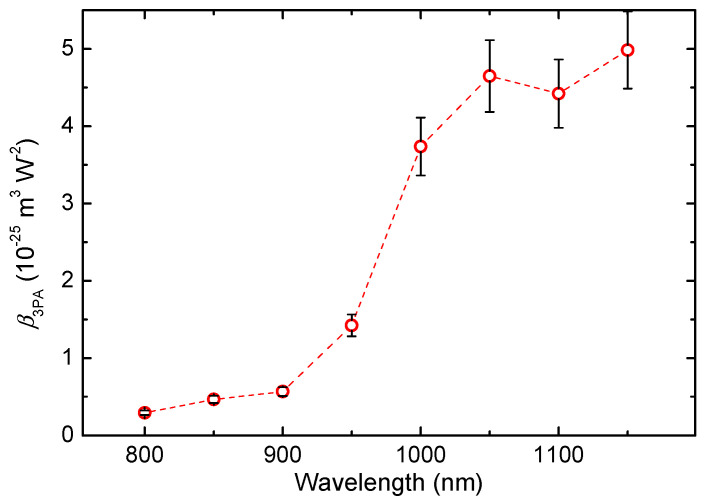
Dependence of 3PA coefficient at low energy measurements using probe pulses of different wavelengths.

Despite the 1150 nm wave (1.08 eV) being slightly under the 3PA threshold for the QDs band gap (3.47 nm), the nonlinear absorption effect becomes stronger when compared with the 950 nm wave. The relative 3PA coefficient values at different wavelengths are shown in Figure 3. It can be seen that a notable increase in nonlinear absorption was observed at wavelengths above 950 nm. The oddity that after the photon energy starts to drop under the 1.15 eV the nonlinear absorption effect continues to increase can be explained, as already mentioned, by the involvement of the higher-order nonlinear absorptive process like four-photon absorption, as it was demonstrated during the explanation of the Ψ(*I*) dependence for 1150 nm (Figure 2b). Another possible reason could be that the real part of permittivity for ITO QDs drops with approaching the ENZ wavelength of 1550 nm for thin ITO films, as it was shown in Ref. [14]. 

### 3.2. Kerr Effect-Induced Refractive Nonlinearities

**Figure 4:**
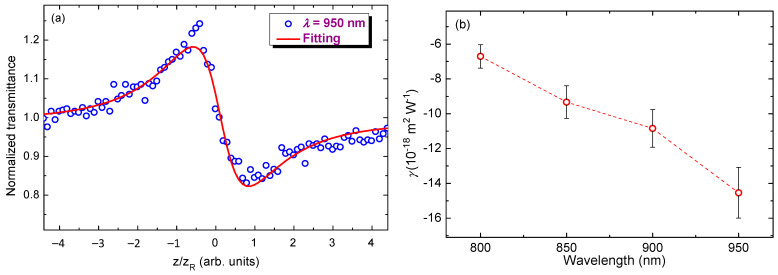
(**a**) Closed-aperture Z-scan measurements of ITO QDs solution using the 950 nm, 0.95 nJ pulse energy. (**b**) Spectral dependence of *γ*.

**Figure 5:**
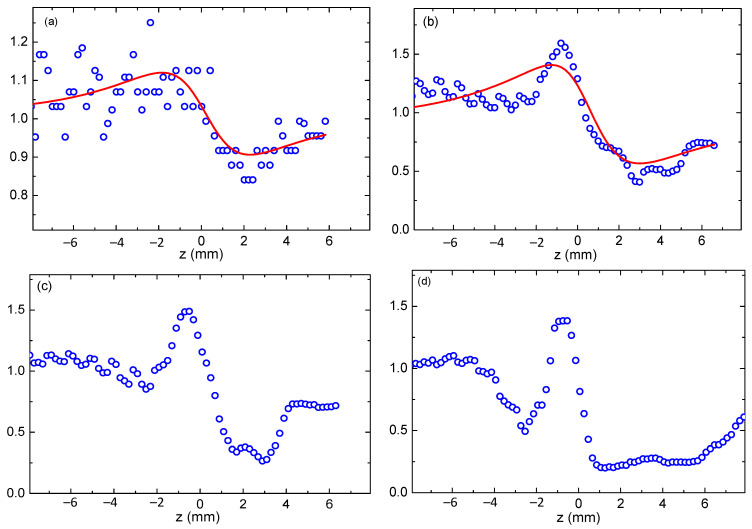
Upper panels: closed-aperture measurements at different energies of 1050 nm radiation and theoretical fittings. (**a**) 0.8 nJ, (**b**) 8.7 nJ. Bottom panels: closed-aperture measurements at approximately the same energies of 1000 nm and 1100 nm radiation. (**c**) 1000 nm, 8.3 nJ, (**d**) 1100 nm, 9.2 nJ.

The Kerr-related refractive nonlinearity was characterized in the 800–1150 nm wavelength range similar to the analysis of the 3PA effect along the broad spectral range. At lower wavelengths, the closed-aperture measurements corresponded to the classical model. The only deviation from the expected results was that Rayleigh length (*z_R_*) calculated from Figure 4a was ~1.2 times larger (1.4 mm) compared to the measured value (1.2 mm). The former value of *z_R_* was deduced using the relation between the distance in peak–valley (ΔZ) in the closed-aperture curve (2.5 mm, Figure 4a) and Rayleigh length (ΔZ ≈ 1.7 × z_R_, [18]) in the case of pure Kerr effect. The closed-aperture data follow this trend up to 950 nm. The induced phase change has a linear dependence on the laser power. We calculated the Kerr coefficient in the 800–950 nm range (Figure 5b) using Equations (2) and (3). The Kerr-induced nonlinear refractive index showed an increase in this parameter from −6.7 × 10^−18^ to −1.5 × 10^−17^ m^2^ W^−1^. The growth of the nonlinear refractive index followed the rule when all studied characteristics of ITO QDs occasionally increased while moving toward the longer-wavelength region.

At longer wavelengths (1000–1100 nm), we observed a significant distortion in closed-aperture data. This effect depended on the laser power. One can compare two scans using 1050 nm radiation of different pulse energies (0.8 and 8.7 nJ, Figure 5a,b). Another example of comparative measurements at 1000 and 1100 nm wavelengths of pump pulses at energies higher than 1 nJ is shown in Figure 5c,d. The possible explanation of such distortions of the closed-aperture curves with regard to the “normal” shape of Z-scan dependence like the one shown in Figure 5a could be the involvement of the higher-order Kerr-related NLO response. One can deduce from the comparison of Figure 5a,b that the contribution of the negative fifth-order Kerr effect starts to play a significant role, as the peak–valley distance becomes narrower than for the base case observed at smaller intensities (powers) of the probe pulses when either lower-order Kerr nonlinearity or thermal effect play the dominant role. 

In the case of relatively high-power 1000 and 1100 nm pulses, the closed-aperture shape becomes even more complex (Figure 5c,d). Notice that previously the demonstrations of involvement of the higher-order Kerr effect have been reported in the case of shorter wavelengths, i.e., close to the absorption bands of the studied materials [22,23]. Particularly, studies of CS_2_ have shown similar shapes of closed-aperture Z-scans in the case of wavelengths below 500 nm [24]. In the case described in Ref [24], the shift between NLO effects was due to the two-photon absorption-induced generation of the excited states at higher photon energies. In our case, the higher-order effects start to dominate at lower photon energies (i.e., at *λ* > 950 nm). 

To the best of our knowledge, no transition of these processes in ITO has been reported in the near-infrared region. One of the possible explanations, for both the growth of the Kerr effect in the longer-wavelength region (800 nm–950 nm range) and the anomalies in closed-aperture measurements (1000–1100 nm range), could be a decrease in refractive index when approaching the ENZ point. As the order of the NLO effect increases, the effect’s efficiency correlation to respective susceptibility becomes stronger. 

The growth of laser intensity led to the appearance of the influence of fifth-order nonlinearity. As one can see, with the growth of input irradiance (close to the focal point), a peak–valley picture appears at the central parts of the *Z*-scan (Figure 5c,d). Those curves resemble two pairs of peaks and valleys, and they are symmetric from the origin. These observations show that the signs of third- and fifth-order nonlinear refractions are the same (negative). There is no transition from self-defocusing to self-focusing that occurs when the sample moves from far to close to the focal point. This phenomenon can be, to some extent, attributed to the thermal effect because the short pulses were used at a high repetition rate, thus allowing the thermal effect. It is known that the heating of the solvent will change its refractive index and cause thermal defocusing. 

Additionally, pulse break-up and beam filamentation at high intensities would cause a phase shift and complex closed-aperture *Z*-scan behavior. Some instability in the appearance of such closed-aperture *Z*-scans can be expected in the presence of the above-mentioned effects. Notice that these complex *Z*-scans appeared each time during our repeated measurements and were reproducible from one set of experiments to another. Also, our laser intensity was not sufficient for the beam filamentation. 

Approximately similar processes were reported previously in the case of carbon disulfide using 110 fs, 795 nm radiation [25]. This phenomenon also was observed in various media [26,27,28,29,30,31] and was analyzed in [32,33]. In most cases, the signs of the third- and fifth-order Kerr nonlinearities were opposite, contrary to the present studies when both nonlinearities demonstrated self-defocusing. 

### 3.3. Third Harmonic Generation in the Thin Films Containing ITO QDs

**Figure 6:**
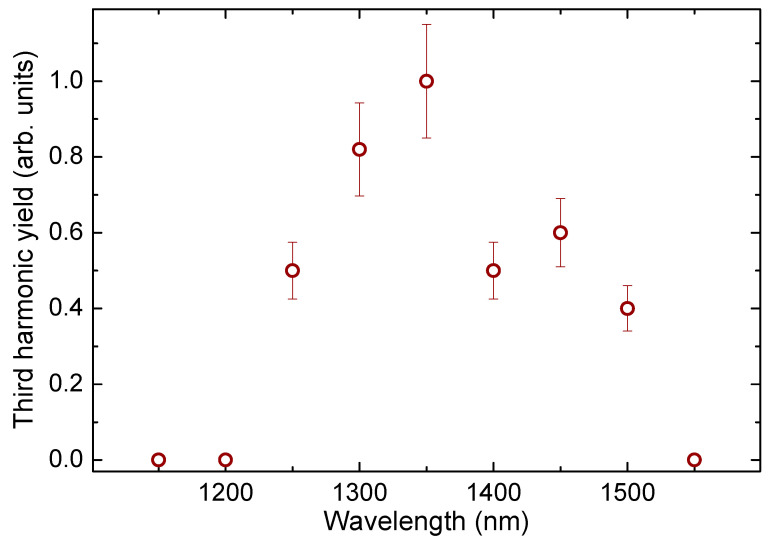
THG conversion efficiency as a function of the probe pulse wavelength.

Finally, we show the results of our studies of the lowest-order nonlinear optical process leading to the generation of the third harmonic of probe radiation in ITO QD thin films. The 3HG was measured in the spectral range between 1100 nm and 1550 nm with a 50 nm step. All measurements were carried out at the same laser power. The relative 3HG intensities in this spectral range are shown in Figure 6. The highest conversion efficiency was observed at *λ* = 1350 nm. In the literature, the ENZ wavelength for ITO thin films varies from 1280 nm [34] to 1553 nm [14] depending on film thickness and fabrication procedure. Surprisingly, this spectral range coincided with the maximal yields of third harmonic generation. Currently, there are no similar measurements of ITO QDs to use as a reference in the literature. 

These studies have shown high efficiency in generating the third harmonic of a tunable laser with pulse duration of 150 fs in the thin (150 nm) films containing ITO quantum dots and deposited on the substrates of fused silica with a thickness of 1 mm. One of the motivations of these 3HG experiments was a search for the potential use of thin films containing similar quantum dots to convert the frequency of laser radiation into the extreme ultraviolet region of the spectrum. It can be assumed that the high efficiency of conversion to a low-order harmonic is related to the high efficiency of higher-order harmonics in small-sized structures. The analysis of the ability of the studied small-sized particles to transform the wavelength of the laser field using a nonlinear optical process of a lower order, such as 3HG, may allow us to make a conclusion about the potential of their application for the generation of higher-order harmonics. 

Third harmonic generation under these conditions was also observed in pure plates of fused quartz glass. The intensity of the third harmonic from a plate of pure glass was three to six times less than in the case of a plate on which a thin film of quantum dots was deposited. Note that the ratio of the thicknesses of the plate of fused quartz glass and films was 1 mm/150 nm ≈ 7000. Accordingly, it was estimated that the third harmonic generated by the thin film was more than 10^8^ times larger than that from a substrate of similar thickness taking into account that the output of harmonics should increase quadratically with increasing thickness of the medium. The absolute value of the THG conversion efficiency in the film used at the wavelength of laser radiation (1350 nm) was estimated to be ~10^−5^. The relatively high conversion efficiency in such a thin film can be explained by the influence of the quantum confinement effect in the case of small-sized particles, such as quantum dots, when the local field can lead to a stronger nonlinear optical interaction with the laser field.

## 4. Discussion

Here, we consider the physics behind the growing absorbance of ITO QDs in IR regions (Figure 1c). The characteristic curve of absorbance of ITO molecules reported in previous publications (for example, [15]) shows a small absorbance close to zero up to wavelengths above 1000 nm, contrary to the case of ITO quantum dot-containing suspension. The reason for the distinction of the absorbance of ITO molecules and ITO QDs and NPs in the longer-wavelength region is as follows.

The characteristic feature of the aggregated structures is the presence of the absorption band attributed to a Mie resonance peak in these tiny species. Depending on the dimensions and specific features of studied small-sized structures, Mie resonance can appear in a broad spectral range (from the deep UV up to the near-IR wavelength region). Regarding the samples examined in the present work, we would like to refer to the study of Bae et al. [16], which has shown that the absorption spectrum of the small-sized aggregates of ITO was notably modified once they considered the species of different sizes compared with the films contained the molecules of ITO. This difference was manifested in the IR range, which showed significant growth of absorbance of the ITO nanoparticle suspensions. Moreover, this spectrum was significantly changed with a decrease in the nanoparticle sizes. In their studies of the two groups of ITO NPs, the broad Mie resonance peak was at 2005 nm for 30 nm ITO NPs and at 1570 nm for 9 nm ITO NPs, respectively.

Their studies have confirmed an appearance of the absorbance of ITO NPs in the IR range compared with the molecular ITO. Moreover, they have demonstrated a shift of the maximum absorbance towards the shorter-wavelength range in the case of the smaller-sized NPs (9 nm). Notice that, for the latter species, the absorbance started to grow already for the longer-wavelength region of the visible spectrum. In our case (QDs of the sizes of 4 nm), the Mie resonance has to be also positioned at the shorter-wavelength region of IR. Correspondingly, the absorbance should show the increase starting from the visible range, which has been demonstrated in Figure 1c. Those absorbance measurements were limited by 1100 nm. 

**Figure 7:**
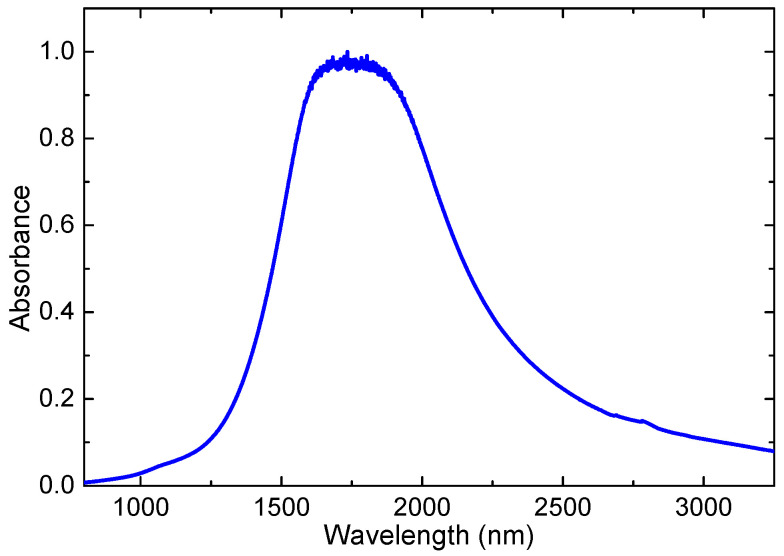
Absorption spectrum of diluted ITO QD suspension in the IR (800–3200 nm) spectral range.

Thus, the physics behind the higher absorbance in the near IR region with regard to the molecular indium–tin oxide suspensions and films can be attributed to the specific properties of the small-sized structures of ITO (4 nm) leading to the appearance of a strong and broad Mie resonance centered in the IR range above 1000 nm. To clarify this issue and demonstrate the appearance of the Mie resonance peak in the case of our samples, we measured the absorption of our diluted ITO QD suspension using the IR spectrometer and observed that a notable growth of this parameter peaked at around 1710 nm (Figure 7). 

The question may arise on the possibility of the application of this peculiarity of ITO QDs in the IR region. Notice that, currently, the use of bulk and molecular ITO is mostly concentrated in the visible range (flat-panel displays, smart windows, polymer-based electronics, thin-film photovoltaics, glass doors of supermarket freezers, architectural windows, coatings that are anti-reflective, and for liquid crystal displays and electroluminescence, where the thin films are used as conducting, transparent electrodes, etc.). The relatively high absorbance of studied QDs in the near IR range does not prevent their use in the abovementioned and other areas of applications. The use of ITO NPs in the near IR range is currently limited to a few areas such as gas sensors and infrared-reflecting coatings (hot mirrors) for the automotive industry. Meanwhile, the Mie resonances in the IR, which are a rare case among the most studied small-sized species, can find applications in nonlinear optics such as the enhancement of the nonlinear optical response of materials under the action of near-IR laser sources. Regarding our finding of three-photon absorption, this specific nonlinear optical process can find application in optical limiting in the near-IR region, optical switches, and optoelectronic devices. 

As for the high absorbance in the UV range (Figure 1c), this is a standard feature of both molecular and NP/QD ITO suspensions and films. The reported band gaps of those species vary between 3.23 eV [11], 3.73, and 3.97 eV for the nano-colloid and the film of ITO [15] and 3.47 eV (our results).

The reason behind the limitation of 3HG studies in the range of 1100 to 1550 nm is as follows. We used similar intensities of the tunable near-IR femtosecond pulses during those experiments to analyze the third harmonic yield at equal conditions of the experiment. The femtosecond laser source used in these studies allowed the tuning of the wavelength of pulses in a broad range (300–1700 nm). The maximal yield of laser emission in this spectral range was at ~1300 nm. Correspondingly, at lower and higher wavelengths, the energies of femtosecond pulses were smaller than at 1300 nm and rapidly dropped. The shortest wavelength of driving pulses during 3HG experiments (1150 nm) still allowed the generation of the third harmonic. The same can be said about the longest wavelength (1550 nm). The pulse energies at these two wavelengths were maintained at the same energy during 3HG studies. Correspondingly, the measurements at other wavelengths in this spectral range were carried out by decreasing the pulse energy down to those of 1150 and 1550 nm pulses. The described procedure allowed for the maintenance of similar conditions during 3HG in this spectral region. 

Fortunately, the used spectral range is of special interest since the epsilon-near-zero wavelength for ITO NPs lies somewhere in this region. This spectral range also surprisingly coincided with the area of the maximal yield of the third harmonic. An additional peculiarity is the position of the Mie resonance peak for the ITO QDs, which was close to the wavelengths of laser pulses at which we observed the maximal yield of the third harmonic. One of the possible assumptions for the enhanced yield of the generated harmonic in this spectral region was the involvement of the resonance effect on the growth of the nonlinear susceptibility responsible for this process.

Similarly, the reasons for the limitation of the spectral range (800–950 nm) of Kerr-induced nonlinear refractive index studies were as follows. One can assume that taking into account our wavelength-dependent measurements of the nonlinear refractive index (Figure 4b), further growth of the wavelength of laser pulses above 950 nm can lead to an increase in the nonlinear refractive index. Probably, similarly to the 3HG and 3PA measurements, this parameter also could be maximized in the 1200 nm or longer wavelength region. However, the obtained closed-aperture Z-scans in the region above 950 nm could not be fitted by a standard procedure, thus preventing the correct calculation of the nonlinear refractive index at the wavelengths higher than 950 nm. Additionally, as we mentioned, at the 1000–1100 nm range, a significant distortion of the closed-aperture Z-scans by the involvement of the higher-order refractive nonlinearity (Figure 5) also prevented determining the lower-order nonlinear refractive index. The shorter wavelength probe pulses (λ < 800 nm) were also, to some extent, phase-distorted, thus leading to the Z-scans, which could not be fitted by the theory. 

Below, we address the significance of the studies of 3PA, 3HG, and Kerr effect in ITO QDs. A study of the Kerr effect in ITO QDs using femtosecond pulses has not been reported so far. Notice that at these short pulses and using the spherical species, no orientational effects take place. Thus, the electronic Kerr effect becomes a dominant process influencing the nonlinear refraction in these species during propagation of the ultrashort laser pulses. The role of the thermal lens seems still insignificant at the used pulse repetition rate (500 kHz) and pulse energy (few hundred pJ). At this regime of pulse repetition rate, the separation time between laser pulses (2 µs) is still less than the thermal characteristic time, which for liquids is of the order of 50 µs. Correspondingly, the accumulative thermal lens, in that case, can cause negative nonlinear refraction only in the case of strong absorption. Another effect can be caused by the free carriers. In the case of ITO film, the temperature-dependent refractive index is positive, so the accumulative heat can induce a positive thermal lens. The latter effect was not registered since we observed only the negative nonlinear refraction at the laser energies below 1 nJ (Figure 4a). 

Like any higher-order nonlinear optical process, 3PA is a relatively rarely studied effect compared with 2PA. Our studies showed that this high-order nonlinear absorption can be affected by an even larger-order process (four-photon absorption, Figure 2b). This is a rare manifestation of the higher-order nonlinear absorption process, which, to the best of our knowledge, has not been reported so far in any material. 

Third harmonic generation in the QD-contained media can serve as a method allowing for making a judgment about the ability of those QDs in the generation of the higher-order harmonics. Earlier, such an assumption found confirmation in the case of metal sulfide and mercury selenide QDs [35,36,37]. Our studies showed that the ITO QDs can also be used as the media for generation of the high-order harmonics in the extreme ultraviolet range. 

The important novelty of these three steps of studies is the wavelength-dependent analysis of the nonlinear optical parameters of ITO QDs. To the best of our knowledge, there are no studies reported that comprised the wavelength-dependent characterization of different nonlinear optical parameters of QDs. Moreover, an approximate coincidence of the epsilon-near-zero region, Mie resonance, and the spectral region of the maximally enhanced third harmonic, as well as of the growth of the 3PA coefficient, points out the possible relation between these effects. 

To summarize the quantitative findings of our research and compare them with previously reported data, we combine in Table 1 the nonlinear optical characteristics of ITO species (QDs, NPs, thin films) from the present study and a few previous reports. 

It is hard to prove that ITO QDs demonstrate better characteristics than bulk ITO. Each of these species shows its positive features. The current status in the applications of bulk ITO is much better understood than the same of QDs. However, some advanced properties of ITO QDs are already seen in our report. QDs can be used for the generation of high-order harmonics and creation of coherent extreme ultraviolet emission, while bulk ITO is useless in this field. QDs can demonstrate the specific quantum-confinement-related features that can be further tuned by altering QD size (Mie resonance, enhancement of various nonlinear optical parameters at a specific spectral region), while bulk ITO cannot provide such demonstration. 

## 5. Conclusions

We presented the first complex study of ITO QDs’ nonlinear optical properties at variable experimental conditions when the parameter of variations (wavelength of a laser beam) towards some specific region significantly modified different nonlinear optical properties of the studied species. The coincidence of the ENZ, maximal 3PA, and 3HG, alongside the closeness of this spectral range with the Mie resonance peak, points out the interconnections of some of these processes in ITO QDs. Previous studies of ITO did not reveal such wavelength-dependent coincidences between different optical and nonlinear optical characteristics of these quantum dots. 

The Kerr-induced nonlinear refractive index was analyzed along the 800–950 nm range showing an increase in this parameter from −6.7 × 10^−18^ to −1.5 × 10^−17^ m^2^ W^−1^. We discussed the peculiarities in the wavelength-dependent variation of the coefficient of nonlinear absorption responsible for the 3PA in the range of 800–1150 nm. 

The three-photon absorption coefficient at 950 nm was measured to be 1.42 × 10^−25^ m^3^/W^2^. The involvement of the higher-order optical nonlinearities (four-photon absorption and fifth-order nonlinear refraction) at higher intensities and powers of the probe pulses was discussed. 

Third harmonic generation was analyzed in the 1200–1550 nm spectral range, with maximal harmonic yield observed at 1350 nm. The absolute value of 3HG conversion efficiency in the 150 nm thick film at the wavelength of laser radiation (1350 nm) was estimated to be ~10^–5^.

The important novelty of these three steps of studies is the wavelength-dependent analysis of the nonlinear optical parameters of ITO QDs. To the best of our knowledge, there are no studies reported that comprised the wavelength-dependent characterization of different nonlinear optical parameters of QDs. 

## Figures and Tables

**Table 1:** Measurements of nonlinear optical parameters of ITO structures reported in previous [11,14,15,38] and present studies.

Material	λ (nm)	*γ* (m^2^/W)	*β*_3PA_ (m^3^/W^2^)	*β*_2PA_ (cm/W)	Comments
ITO nanoparticles	632.8	1.7 × 10^−11^			CW laser [15]
ITO thin film	1050 1550			−5.0 × 10^−10^ −6.4 × 10^−10^	[14]
ITO thin film	720 900	(1.72 ± 0.17) × 10^−16^ (5.34 ± 0.53) × 10^−16^		(1.05 ± 0.11) × 10^−9^ (1.34 ± 0.13) × 10^−9^	80 fs, 1 MHz [11]
ITO thin film	1550			−6.85 × 10^−9^	200 fs, 1 kHz [38]
ITO quantum dots	800	−(6.7 ± 1.3) × 10^−18^	(0.293 ± 0.048) × 10^−25^		Present study
	850	−(9.3 ± 1.6) × 10^−18^	(0.466 ± 0.097) × 10^−25^		
	900	−(10.8 ± 2.1) × 10^−18^	(0.57 ± 0.16) × 10^−25^		
	950	−(14.5 ± 2.2) × 10^−18^	(1.42 ± 0.14) × 10^−25^		
	1000		(3.74 ± 0.97) × 10^−25^		
	1050		(4.65 ± 0.80) × 10^−25^		
	1100		(4.42 ± 0.54) × 10^−25^		
	1150		(4.98 ± 0.50) × 10^−25^		

## Data Availability

The data that support the findings of this study are available on request from the corresponding author.
